# Rapidly progressive glomerulonephritis due to IgA nephropathy accompanied by collagenofibrotic glomerulopathy. A nephrology picture

**DOI:** 10.1007/s40620-023-01875-7

**Published:** 2024-02-06

**Authors:** Shoko Ochiai, Masao Kikuchi, Koichi Kaikita, Shouichi Fujimoto

**Affiliations:** Division of Cardiovascular Medicine and Nephrology, Department of Internal Medicine, Faculty of Medicine, University of Miyazaki, 5200 Kihara, Kiyotake, Miyazaki, 889-1692 Japan

A 69-year-old Japanese woman was admitted to our hospital with an approximately one-month history of fatigue and generalized pitting edema. Her sister had been on hemodialysis since her 20s, but the underlying disease was unknown. The patient had been diagnosed with renal dysfunction with serum creatinine (SCr) levels of approximately 1.5 mg/dL and proteinuria, but her disease was not progressive and had not been investigated. At referral she exhibited gross hematuria, elevated SCr levels (5.8 mg/dL), nephrotic syndrome, and a weight gain of 10 kg above her usual body weight. There was no marked decrease in complement levels, and test results for antineutrophil cytoplasmic antibody, and anti-GBM antibodies were negative. Renal biopsy showed a membranoproliferative glomerulonephritis (MPGN)-like pattern of all glomeruli (Fig. 1A) with cellular crescent formation in one glomerulus. At the immunofluorescence study, IgA deposition dominated the capillary walls (Fig. 1B) and was positive for the KM55 monoclonal antibody specific to Gd-IgA1 (Fig. 2A). Electron microscopy revealed two alterations: those visualized in the mesangial areas by oolong tea extract staining and electron-dense deposits found in the subendothelial areas (Fig. 1D, E). Congo red staining was negative, while immunohistochemical analysis for type III collagen was positive (Fig. 1C). Serum procollagen III N-terminal propeptide and hyaluronic acid levels were elevated to 602 ng/mL and 138,000 ng/mL, respectively. Based on these findings, the patient was diagnosed with IgA nephropathy (IgAN) with collagenofibrotic glomerulopathy. Temporary hemodialysis was necessary; however, steroid pulse therapy and cyclophosphamide improved the SCr to 2.66 mg/dL and resolved the patient's hematuria. Proteinuria remained at a nephrotic level. Collagenofibrotic glomerulopathy is a rare disease characterized by the accumulation of type III collagen in glomeruli [1]. IgAN is the most common primary glomerulonephritis. Although mesangial IgA deposition is observed in IgAN, IgA deposition in the pericapillary walls correlates with higher proteinuria and crescent formation on biopsy, indicating a poor prognosis for kidney function [2]. In this case, IgA deposition dominated the capillary walls over the mesangial area, while type III collagen deposition occupied the mesangial area, showing a MPGN-like pattern. The abundance of type III collagen in the mesangial area might have facilitated IgA deposition in the capillary walls (Fig. 2B).

**Fig. 1:**
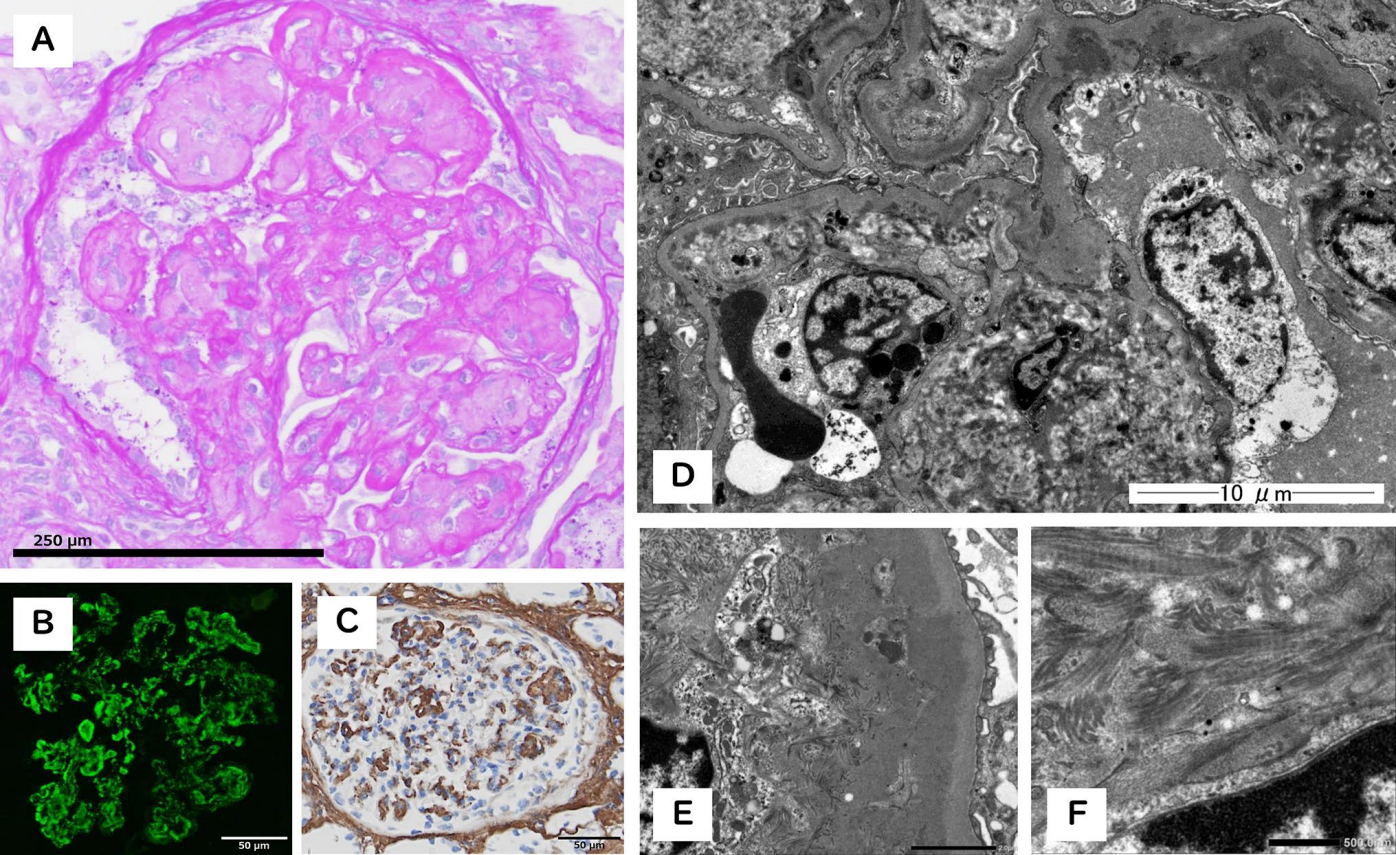
Light microscopic, fluorescence immunohistochemistry, and electron microscopy study findings. **A** PAS staining sections showing enlarged mesangial matrix and segmental lobulation and reduced staining levels in the mesangial area, with glomerular capillaries stained deep red with a “wire loop” lesion appearance. **B** IF study findings: IgA deposition is detected, and the deposition is particularly pronounced in the capillary walls compared with that in the mesangial areas. **C** Type III collagen immunostaining showing positive findings. **D** Electron microscopy study findings. Mesangial matrix expansion and two types of structures in the mesangial area and subendothelial area are observed. **E** Oolong tea extract staining shows fibrous structure-like deposits in the mesangial area and electron-dense deposits in the subendothelial area, respectively. **F** The deposits in the mesangial area show an irregular fibrous structure with random arrangement; transverse band structures with a periodicity of 43–65 nm are observed

**Fig. 2:**
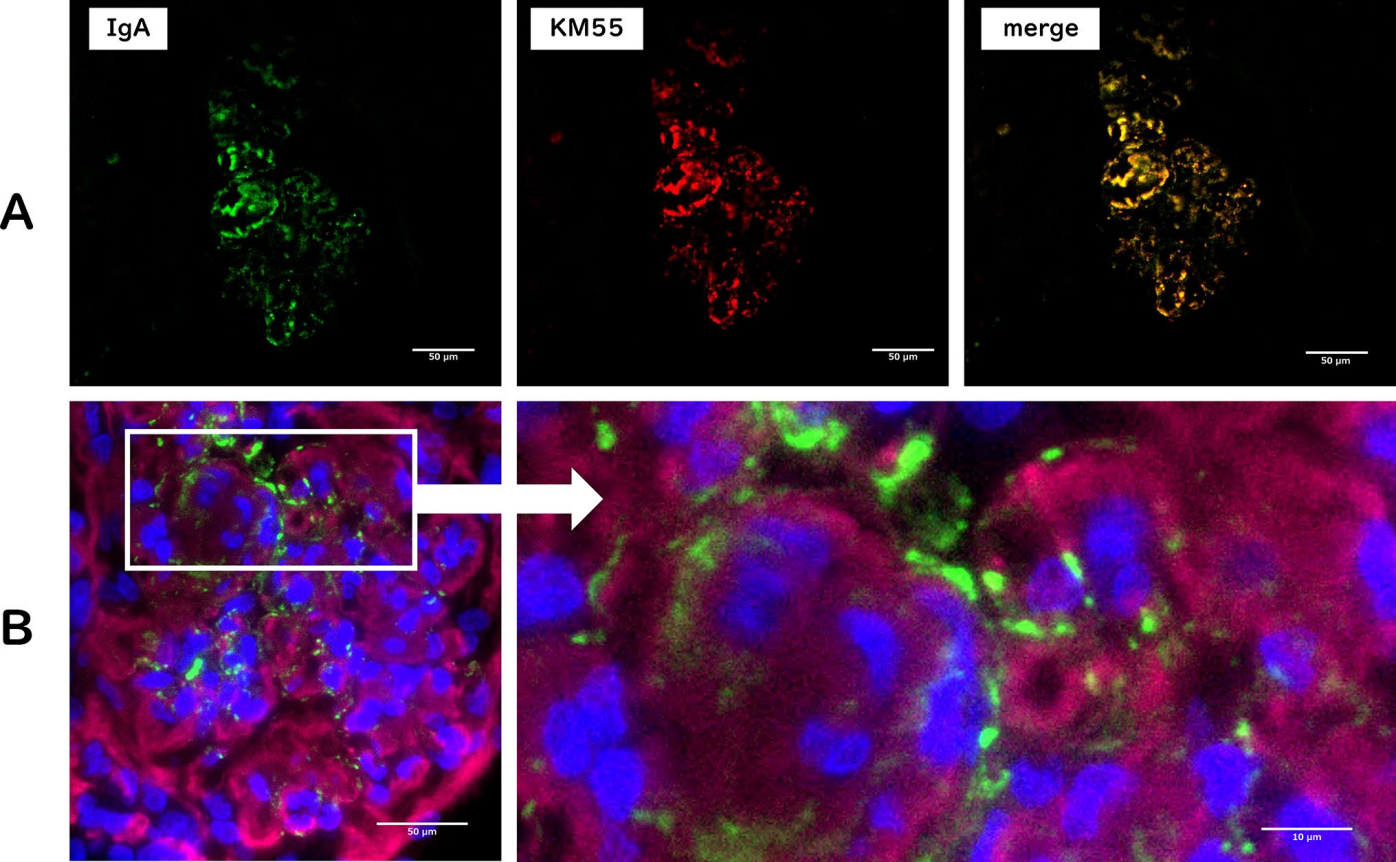
Immunohistochemistry. **A** Double staining with an anti-IgA polyclonal antibody and KM55 monoclonal antibody. GdIgA1, as detected by KM55, matching the location of IgA. **B** blue: DAPI, green: IgA, red: type III collagen. Triple staining reveals that type III collagen occupies the mesangial area, and IgA is observed in the subendothelium, where type III collagen is not deposited

Rapidly progressive glomerulonephritis is a rare clinical manifestation of collagenofibrotic glomerulopathy and IgAN. Accurate interpretation of renal biopsy may improve renal outcomes.

## Data Availability

All data supporting the case are included in the manuscript.
